# Use of antidiabetic medications and risk of chronic obstructive pulmonary disease exacerbation requiring hospitalization: a disease risk score-matched nested case–control study

**DOI:** 10.1186/s12931-020-01547-1

**Published:** 2020-12-02

**Authors:** Meng-Ting Wang, Jyun-Heng Lai, Ya-Ling Huang, Feng-Chih Kuo, Yun-Han Wang, Chen-Liang Tsai, Min-Yu Tu

**Affiliations:** 1 School of Pharmacy, National Defense Medical Center, Taipei, Taiwan; 2 Department of Pharmacy, En Chu Kong Hospital, New Taipei City, Taiwan; 3 Division of Endocrinology and Metabolism, Department of Internal Medicine, Tri-Service General Hospital, National Defense Medical Center, Taipei, Taiwan; 4 Clinical Epidemiology Division, Department of Medicine, Solna, Karolinska Institutet, Stockholm, Sweden; 5 Division of Pulmonary and Critical Care, Tri-Service General Hospital, National Defense Medical Center, Taipei, Taiwan; 6 Department of Health Business Administration, Meiho University, Pingtung, Taiwan; 7 Aviation Physiology Research Laboratory, Kaohsiung Armed Forces General Hospital Gangshan Branch, No.1, Dayi 2nd Rd., Gangshan Dist., Kaohsiung City, 82050 Taiwan; 8 Institute of Medical Science and Technology, National Sun Yat-Sen University, Kaohsiung, Taiwan; 9 Department of Life Sciences, and Ph.D. Program in Translational Medicine, National Chung Hsing University, Taichung, Taiwan

**Keywords:** Antidiabetic agents, Chronic obstructive pulmonary disease, Diabetes mellitus, COPD exacerbation, Observational study

## Abstract

**Background:**

Exacerbation of chronic obstructive pulmonary disease (COPD) severely impacts the quality of life and causes high mortality and morbidity. COPD is involved with systemic and pulmonary inflammation, which may be attenuated with antidiabetic agents exerting anti-inflammatory effects. Real-world evidence is scant regarding the effects of antidiabetic agents on COPD exacerbation. Accordingly, we conducted a disease risk score (DRS)-matched nested case–control study to systemically assess the association between each class of oral hypoglycemic agents (OHAs) and risk of severe COPD exacerbation in a nationwide COPD population co-diagnosed with diabetes mellitus (DM).

**Methods:**

We enrolled 23,875 COPD patients receiving at least one OHA for management of DM by analyzing the Taiwan National Health Insurance claims database between January 1, 2000, and December 31, 2015. Cases of severe exacerbation were defined as those who had the first hospital admission for COPD. Each case was individually matched with four randomly-selected controls by cohort entry date, DRS (the estimated probability of encountering a severe COPD exacerbation), and COPD medication regimens using the incidence density sampling approach. Conditional logistic regressions were performed to estimate odds ratios (OR) of severe COPD exacerbation for each type of OHAs.

**Results:**

We analyzed 2700 cases of severe COPD exacerbation and 9272 corresponding controls after DRS matching. Current use of metformin versus other OHAs was associated with a 15% (adjusted OR [aOR], 0.85; 95% confidence interval [CI] 0.75–0.95) reduced risk of severe COPD exacerbation, whereas the reduced risk was not observed with other types of antidiabetic agents. When considering the duration of antidiabetic medication therapy, current use of metformin for 91–180 and 181–365 days was associated with a 28% (aOR, 0.72; 95% CI 0.58–0.89) and 37% (aOR, 0.63; 95% CI 0.51–0.77) reduced risk of severe COPD exacerbation, respectively. Similarly, 91–180 days of sulfonylureas therapy led to a 28% (aOR, 0.72; 95% CI 0.58–0.90) lower risk, and longer treatments consistently yielded 24–30% lower risks. Current use of thiazolidinediones for more than 181 days yielded an approximately 40% decreased risk.

**Conclusions:**

Duration-dependent beneficial effects of current metformin, sulfonylurea, and thiazolidinedione use on severe COPD exacerbation were observed in patients with COPD and DM.

## Background

Chronic obstructive pulmonary disease (COPD) is a globally prevalent disease with irreversible and persistent airway obstruction, currently affecting at least 380 million people worldwide [1]. According to recent statistics of World Health Organization, more than 3 million patients died of COPD globally in 2015, with a death rate of 5% [2]. Approximately 30% of COPD patients experienced one or more exacerbation events yearly [3], and COPD exacerbations often elicited severe clinical consequences, despite standard COPD treatments. Patients with COPD exacerbation had an inpatient death rate of 4–11% on index hospitalization, and 22–43% of them died within 1 year after discharge [4, 5]. Inhaled long-acting β_2_ agonists, long-acting muscarinic antagonists, and inhaled corticosteroids are the mainstay therapies for managing COPD [6]; however, these medications could not entirely prevent COPD exacerbations [7]. Consequently, development of novel therapies for reducing COPD exacerbations is of great clinical importance.

Diabetes mellitus (DM) frequently coincides with COPD [8], and use of specific hypoglycemic agents (OHAs) may decrease COPD progression due to their anti-inflammatory effect based on preclinical data [9]. Exacerbation of COPD is primarily involved with systemic and pulmonary inflammation [10], which may be attenuated with using several antidiabetic agents [9]. According to in vitro and in vivo studies, metformin, thiazolidinedione (TZD), and dipeptidyl peptidase-4 (DPP-4) inhibitors produce anti-inflammatory effects by decreasing inflammatory biomarker levels [11, 12], activating peroxisome proliferator-activated receptor-γ [13], or improving insulin resistance [14, 15], but several OHAs, such as sulfonylureas, meglitinides, and α-glucosidase inhibitors, have not been fully assessed regarding their effects in inflammation and COPD. Therefore, further investigations are urgently required to assess whether or not each individual type of antidiabetic medications carries a beneficial effect on COPD outcomes.

Only one case–control study and a cohort-study have evaluated the association between risk of COPD exacerbation and use of antidiabetic drugs among COPD patients, which reported a 44% lower risk of COPD-related hospitalizations or emergency visits with metformin use [16] and a 19% reduced risk of COPD exacerbation requiring hospitalization with TZD use [17], respectively. Nevertheless, the two studies may be subject to selection bias, lack of control of important confounding factors, such as pneumonia and lung cancer, and confounding by DM and COPD severity. Additionally, the impact of other types of OHAs on COPD exacerbations has not been examined.

To the best of our knowledge, no study has extensively evaluated the association between risk of COPD exacerbations and each class of OHAs. Accordingly, this study was aimed to assess the association between use of various types of OHAs and the risk of severe COPD exacerbations in patients with COPD and DM.

## Methods

### Study design and data source

We implemented a disease risk score (DRS)-matched nested case–control design because (1) severe COPD exacerbation requiring hospitalization is a rare outcome, (2) multiple classes of OHAs are the exposures of interest, and (3) treatment with OHAs is often changed during the time course of diabetes management. A nested case–control design is reportedly effective for assessing time-varying drug usage and can yield an unbiased rate ratio [18]. Moreover, this design is less affected by selection bias than a traditional case–control study because cases and controls are selected from the same cohort. The adoption of a disease risk score-matched approach was to maintain the balance in the probability of encountering the first severe COPD exacerbation between cases and controls at the baseline.

We analyzed the Taiwan National Health Insurance claims database from January 1, 2000, to December 31, 2015. The National Health Insurance currently covers approximately 99% of the 23 million Taiwanese inhabitants. Specifically, we analyzed the Longitudinal Generation Tracking Database (LGTD) of two million randomly selected beneficiaries, which are reportedly representative of the National Health Insurance beneficiaries in terms of distribution of insurance premium, insured area, and top 10 causes of deaths [19]. The analyzed database contains all claims of de-identified and encrypted demographics, clinical diagnoses, procedures, and prescription-refill records from outpatient, inpatient, and emergency care settings. We also linked the claims database to the National Death Certification Registry using encrypted patient identifiers. This study was exempt from a full review by the Institutional Review Board of Tri-service General Hospital, National Defense Medical Center, Taipei, Taiwan (B-105-14).

### Cohort identification

We identified the study cohort as patients with COPD and initially receiving at least one OHA for management of DM between January 1, 2001, and December 31, 2014. Specifically, patients with COPD were identified if they had two outpatient visits or one inpatient visit for COPD (International Classification of Diseases, Ninth Revision, Clinical Modification [ICD-9-CM] codes 491.xx, 492.xx, and 496.xx) within a year and had received COPD medications. The date of the first COPD-related outpatient visit or the discharge date from a COPD hospitalization during the study period marked the COPD date. Patients with COPD were further required to have the first prescription-refill record of OHAs (metformin, sulfonylureas, TZDs, DPP-4 inhibitors, α-glucosidase inhibitors, and meglitinides) after the COPD date and were additionally required to have two outpatient visits or an inpatient visit for DM (ICD-9-CM code 250.xx) within the year before or after the refill date of the first OHA prescription, which was set as cohort entry date. The employed algorithm for identifying diabetic patients has been reported to be highly accurate [20]. Patients with COPD and DM were additionally required to be aged 20 years or more at cohort entry date.

Patients with the following criteria in the year preceding the cohort entry date were excluded: (1) < 1 year of continuous health insurance enrollment, (2) any diagnosis of lung cancer (ICD-9-CM codes 162.xx, 163.xx, and 165.xx), and (3) use of metformin for the treatment of polycystic ovarian syndrome (ICD-9-CM code 256.4x) and gestational diabetes (ICD-9-CM code 648.8x). We excluded patients with lung cancer to avoid capturing COPD exacerbation from cancer progression and those with polycystic ovarian syndrome or gestational diabetes because metformin is the suggested medication for both conditions. Additionally, in order not to observe a subsequent COPD exacerbation from the prior exacerbation at baseline, patients with severe COPD exacerbations (defined below in case identification) occurring during the 30 days prior to cohort entry were excluded. Users of antidiabetic glucagon-like peptide-1 receptor agonists were excluded as well due to limited size. The study cohort was followed up from cohort entry until occurrence of the first severe COPD exacerbation requiring hospitalization (defined below), discontinuation of the National Health Insurance program, death, or December 31, 2015.

### Case identification and control selection

Cases of severe COPD exacerbations were defined as patients who had an inpatient visit with a primary diagnosis of COPD (ICD-9-CM codes 491.xx, 492.xx, 496.xx) or those who had a first hospital admission for acute respiratory failure (ICD-9-CM codes 518.81, 518.82, 518.84, and 786.09) and COPD as a primary and secondary cause, respectively, during follow-up. We considered severe exacerbation as a primary outcome because it is one of the most serious respiratory events that could impact COPD patients’ quality of life and increase mortality substantially [21, 22]. The diagnosis of acute respiratory failure was taken into account when measuring severe exacerbations because it is the worst prognosis following COPD exacerbation [23]. The algorithms for identifying severe COPD exacerbation has been employed previously [24, 25]. The index date was defined as the date of the first occurrence of severe COPD exacerbation.

Each case was individually matched with four randomly-selected controls by cohort entry date (± 180 days), DRS (± 0.01) that predicted occurrence of the first severe COPD exacerbation, and COPD medication regimens (categorized as nonuse, dual therapy of LABA/LAMA or LABA/ICS combination, triple therapy of LABA/LAMA/ICS, and other regimens) among risk sets. The DRS was estimated for each cohort member from a logistic regression model, considering all confounders measured at baseline and deciles of duration from the COPD date to cohort entry. Employing DRS as a matching criterion in a nested case–control design reportedly yields a better statistical precision than using multiple individual factors [26]. We adopted DRS and COPD medication regimen complexity as the matching criteria to maintain the balance in exacerbation risk at baseline and in severity of COPD between cases and controls, respectively.

### Measurement of exposure

All prescription-filling records of OHAs from outpatient, inpatient, and emergency care settings were examined preceding the index/event date for both cases and controls. We classified cases and controls as current users and current nonusers for each class of non-insulin antidiabetic medications, and compared current use versus current nonuse of each class of antidiabetic agents regarding the risk of severe COPD exacerbation. Specifically, current use was defined as the end of the latest prescription date of the OHAs falling within 30 days preceding the index date/event date. Current use of OHAs in each class was further classified according to duration of therapy (≤ 30, 31–90, 91–180, 181–365, and > 365 days) and average daily dose levels (< 0.5 defined daily dose [DDD], 0.51–1 DDD, and > 1 DDD). To address the complexity of antidiabetic medication regimens and confounding by indication bias, we further compared current use of each class of antidiabetic medications with current use of other class of antidiabetic agents in monotherapy, dual therapy and triple therapy, respectively.

### Measurement of covariates

We considered proxies of COPD severity and DM severity, comorbidities and co-medications that may be related to use of OHAs or occurrence of severe COPD exacerbation. Measures of proxy indicators of COPD severity included hospitalization for COPD (0, 1, ≥ 2 visits), emergency room (ER) visits for COPD (0, 1, ≥ 2 visits), and outpatient COPD exacerbation (0, 1, ≥ 2 visits), defined as an outpatient visit with a primary diagnosis of COPD accompanied by use of oral corticosteroids or antibiotics for 3–14 days. The COPD proxies in outpatient and ER settings were measured within the year preceding the cohort entry date, and, because severe COPD exacerbation requiring hospitalization in the 30 days prior to cohort entry was set as an exclusion criterion, we additionally measured COPD exacerbation requiring hospitalization within the 31–365 days before cohort entry date. We considered diabetic severity, including adapted diabetes complications severity index, insulin (short-acting and long-acting insulin) use, metabolic acidosis, and regimens of OHAs (monotherapy, dual therapy, triple therapy or more). To address immortal time bias, the duration between COPD date and cohort entry date was considered as well. Other potential confounders related to OHA use or severe COPD exacerbations were measured, such as sex, age, individual COPD medications, pulmonary disease, cardiovascular disease, and receipt of influenza and pneumonia vaccines. All of the confounders were first measured in the year preceding cohort entry (except for the mentioned COPD-related hospitalization) and were included in the multivariate logistic regression to estimate the DRS. We additionally measured all covariates preceding the index date. Comorbidities, COPD severity indicators, aDSCI, metabolic acidosis, and pneumonia and influenza vaccinations were measured in the year preceding the index date, and co-medications and other DM severity indicators were measured in the 6 months before the index date.

### Statistical analysis

We employed standardized difference to compare clinical and demographic factors between cases and controls, using the cut-off value of 0.1 as meaningful imbalanced distributions between comparison groups [27]. We also employed conditional logistic regression models to estimate odds ratios (ORs) of severe COPD exacerbation requiring hospitalization associated with use of non-insulin antidiabetic medications. Two models of statistical adjustment were conducted. First, we adjusted for all covariates with standardized difference > 0.1 (model 1). Second, we additionally adjusted for different regimens of diabetic therapy in the 180 days prior to the index date (model 2). We tested linear trends in the dose-response or duration-response associations. Data cleaning and statistical analyses were performed using SAS 9.4 (SAS Institute) and STATA 14.0 (STATA Corp) with two-sided *p* values less than 0.05 as statistically significant.

### Sensitivity and subgroup analysis

Multiple additional analyses were conducted. First, to increase the accuracy of the employed algorithms for identifying COPD patients, we restricted the study population to those who underwent spirometry testing. Second, to avoid the influence of previous severe COPD exacerbations, we extended the 30-day period to a 1-year period preceding cohort entry to exclude patients with severe exacerbations. Third, to eliminate cardiogenic causes of COPD exacerbation, we excluded patients diagnosed with heart failure in the year preceding the index date. Fourth, to alleviate the impact of diabetic severity, we excluded any refill record of insulin prescriptions at baseline and during follow-up. Fifth, we used different statistical models, including traditional multivariable logistic regressions, adjusting for different classes of other OHAs during follow-up, and adjusting for all covariates. Sixth, we performed stratified analyses by the adapted diabetes complications severity index (0, 1–2, 3–4, and ≥ 5), type of care for COPD during follow-up (outpatient-related, emergency-related, and no exacerbations), presence of asthma, and use of systemic corticosteroids. Finally, we performed a rule-out method to assess the impact of unmeasured confounding on the positive findings in the primary analyses.

## Results

**Fig. 1:**
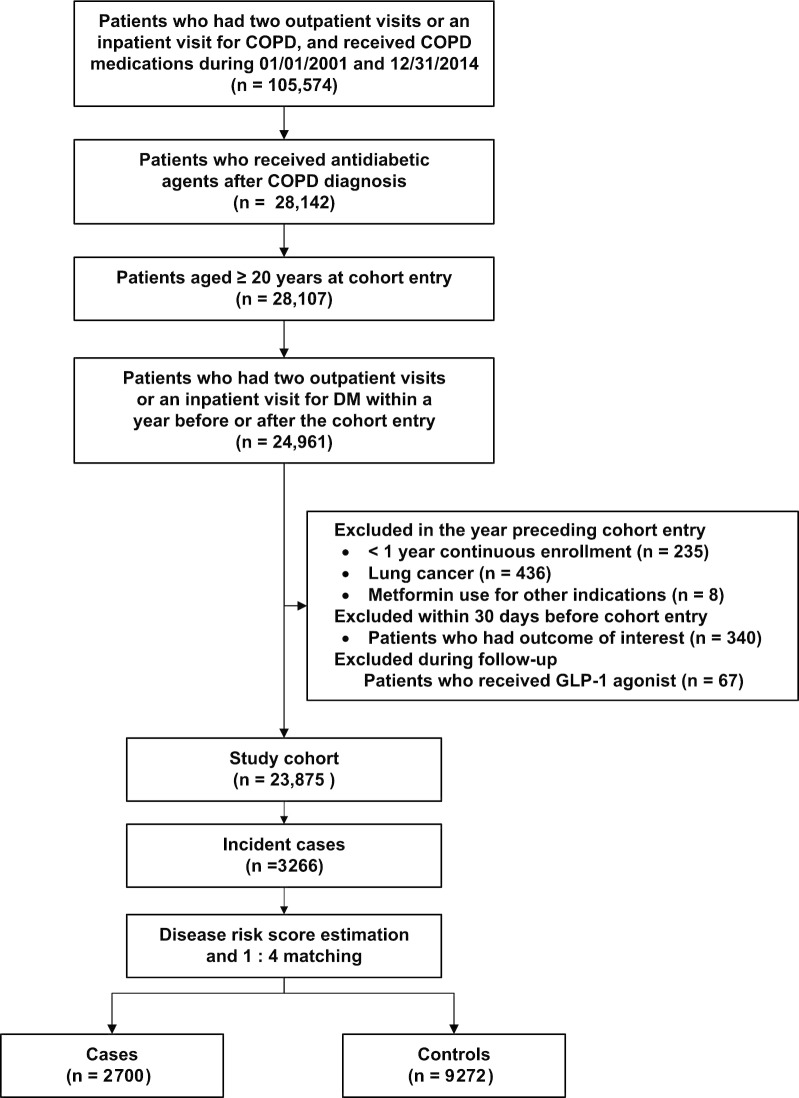
Flow diagram outlining the selection of study cohort, cases, and controls. COPD, chronic obstructive pulmonary disease; DM, diabetes mellitus; glucagon-like peptide-1, GLP-1

The eligible study cohort comprised 23,875 COPD patients (mean age, 69.1 years; 59.4% men) co-diagnosed with diabetes and receiving OHAs (Fig. 1). The study cohort was followed for a mean of 5.2 years, contributing to 124,325 person-years. During the follow-up, 3266 patients with severe COPD exacerbation were identified, with an incidence rate of 2.62 per 100 person-years. After DRS matching, we included 2700 cases and 9272 randomly-selected matched controls.

Overall, the baseline characteristics were balanced between cases and controls, despite a few imbalanced covariates between the two groups (Table 1). During the follow-up, most factors remained balanced between cases and controls. However, cases had higher numbers of COPD exacerbations with ER visits, presence of stroke, heart failure, and pneumonia, as well as receipt with co-medications, such as short-acting insulin, diuretics, antiarrhythmic agents, and systemic corticosteroids. Controls had a higher proportion of hyperlipidemia and use of statins, nonsteroidal anti-inflammatory drugs, and vaccines than cases.

**Table 1:** Clinical characteristics of cases and matched controls

Characteristics	At Baseline, No. (%)^a^			During Follow-up, No. (%)^c^		
	Cases (n = 2700)	Controls (n = 9272)	SD^b^	Cases (n = 2700)	Controls (n = 9272)	SD^b^
Age, mean ± s.d	73.2 ± 10.3	71.3 ± 10.1	0.193	72.8 ± 10.1	71.9 ± 9.9	0.193
Sex, male No. (%)	1800 (66.7)	6210 (67.0)	0.007	1912 (69.3)	7364 (70.1)	0.007
Periods from the first COPD visit to the initial use of antidiabetic agents, days (mean ± s.d*.*)	519 ± 922	435 ± 826	0.052	NA	NA	NA
DM severity indicators, No. (%)						
aDCSI						
0	742 (27.5)	2930 (31.6)	0.090	764 (28.3)	3074 (33.2)	0.105
1–2	1061 (39.3)	3778 (40.8)	0.030	1024 (37.9)	3622 (39.1)	0.023
3–4	626 (23.2)	1846 (19.9)	0.080	633 (23.4)	1845 (19.9)	0.086
≥ 5	271 (10.0)	718 (7.7)	0.090	279 (10.3)	731 (7.9)	0.085
Insulin						
Nonusers	1786 (66.2)	6634 (71.6)	0.117	1801 (66.7)	6864 (74.0)	0.161
Short acting only	831 (30.8)	2366 (25.5)	0.117	754 (27.9)	1899 (20.5)	0.174
Long acting	83 (3.1)	272 (2.9)	0.008	145 (5.4)	509 (5.5)	0.005
Regimens of oral hypoglycemic agents^d^						
Nonusers	0	0		333 (12.3)	919 (9.9)	0.077
Monotherapy	1376 (51.0)	4958 (53.5)	0.050	940 (34.8)	2939 (31.7)	0.066
Dual therapy	944 (35.0)	3286 (35.4)	0.010	900 (33.3)	3508 (37.8)	0.094
Triple therapy or more	380 (14.1)	028 (11.1)	0.090	527 (19.5)	1906 (20.6)	0.026
Metabolic acidosis	9 (0.3)	21 (0.2)	0.020	21 (0.8)	22 0.2)	0.076
New users of oral hypoglycemic agents	1589 (58.9)	5744 (62.0)	0.063	NA	NA	NA
COPD severity indicators, No. (%)						
No. of inpatient visit for COPD exacerbations^e^						
0	2589 (95.9)	9032 (97.4)	0.085	NA	NA	NA
1	85 (3.2)	201 (2.2)	0.061	NA	NA	NA
≥ 2	26 (1.0)	39 (0.4)	0.065	NA	NA	NA
No. of ER visit for COPD exacerbations						
0	2590 (95.9)	9017 (97.3)	0.073	2488 (92.2)	8940 (96.4)	0.185
1	85 (3.2)	221 (2.4)	0.047	149 (5.5)	304 (3.3)	0.109
≥ 2	25 (0.9)	34 (0.4)	0.070	63 (2.3)	28 (0.3)	0.179
No. of OPD visit for COPD exacerbations^f^						
0	2284 (84.6)	7876 (84.6)	0.010	2252 (83.4)	8129 (87.7)	0.121
1	296 (11.0)	1013 (11.0)	0.001	289 (10.7)	768 (8.3)	0.083
≥ 2	120 (4.4)	383 (4.4)	0.015	159 (5.9)	375 (4.0)	0.085
Types of COPD regimens						
Nonusers	894 (33.1)	3,040 (32.8)	0.007	360 (13.3)	1,306 (14.0)	0.022
Triple therapy	15 (0.6)	33 (0.4)	0.030	15 (0.6)	15 (0.2)	0.066
Dual therapy	224 (8.3)	722 (7.8)	0.019	305 (11.3)	719 (7.8)	0.121
Others	1567 (58.0)	5477 (59.1)	0.021	2020 (74.8)	7,232 (78.0)	0.075
Comorbidities, No. (%)						
CV diseases						
Hypertension	1951 (72.4)	6572 (70.9)	0.033	1906 (70.6)	6720 (72.5)	0.042
Coronary artery disease	895 (33.2)	3009 (32.5)	0.015	853 (31.6)	2873 (31.0)	0.013
Stroke	826 (30.6)	2122 (22.9)	0.175	911 (33.7)	2416 (26.1)	0.168
Hyperlipidemia	636 (23.6)	2390 (25.8)	0.052	581 (21.5)	2695 (29.1)	0.174
Heart failure	488 (18.1)	1387 (15.0)	0.084	625 (23.2)	1518 (16.4)	0.171
Arrhythmia	387 (14.3)	1193 (12.9)	0.043	427 (15.8)	1166 (12.6)	0.093
Peripheral vascular disease	171 (6.3)	551 (5.9)	0.016	168 (6.2)	540 (5.8)	0.017
Rheumatic heart disease	46 (1.7)	122 (1.3)	0.032	51 (1.9)	151 (1.6)	0.020
Pulmonary embolism	7 (0.3)	15 (0.2)	0.021	10 (0.4)	21 (0.2)	0.026
Pulmonary disease						
Acute bronchitis	1090 (40.4)	4107 (44.3)	0.079	905 (33.5)	3188 (34.4)	0.018
Asthma	819 (30.3)	3092 (33.4)	0.065	897 (33.2)	2729 (29.4)	0.082
Pneumonia	804 (29.8)	2386 (25.7)	0.090	972 (36.0)	2598 (28.0)	0.172
Influenza	193 (7.2)	780 (8.4)	0.047	109 (4.0)	500 (5.4)	0.064
Mental disease						
Dementia	264 (9.8)	523 (5.6)	0.156	342 (12.7)	709 (7.7)	0.167
Depression	153 (5.7)	476 (5.1)	0.024	148 (5.5)	433 (4.7)	0.037
Schizophrenia	15 (0.6)	49 (0.5)	0.004	15 (0.6)	43 (0.5)	0.013
Chronic liver disease	322 (11.9)	1214 (13.1)	0.035	257 (9.5)	1004 (10.8)	0.043
Cancer	311 (11.5)	1011 (10.9)	0.019	345 (12.8)	1279 (13.8)	0.030
Sepsis	227 (8.4)	424 (4.6)	0.156	390 (14.4)	769 (8.3)	0.195
Chronic renal disease	216 (8.0)	538 (5.8)	0.087	347 (12.9)	917 (9.9)	0.093
GERD	144 (5.3)	397 (4.3)	0.049	212 (7.9)	617 (6.7)	0.046
Parkinsonism	132 (4.9)	303 (3.3)	0.082	148 (5.5)	365 (3.9)	0.073
Co-medication, No. (%)						
CV medication						
Diuretics	1771 (65.6)	5709 (61.6)	0.084	1755 (65.0)	5425 (58.5)	0.134
Calcium channel blockers	1724 (63.9)	5766 (62.2)	0.034	1616 (59.9)	5275 (56.9)	0.060
Antiplatelets	1688 (61.8)	5508 (59.4)	0.049	1465 (54.3)	4722 (50.9)	0.067
Angiotensin-converting enzyme inhibitors	874 (32.4)	3113 (33.6)	0.026	579 (21.4)	2014 (21.7)	0.007
Angiotensin receptor blockers	849 (31.4)	2754 (29.7)	0.038	938 (34.7)	3479 (37.5)	0.058
Nitrates	665 (24.6)	2011 (21.6)	0.072	617 (22.9)	1773 (19.1)	0.092
β-blockers						
Non-CV selective	641 (23.7)	2088 (22.5)	0.029	418 (15.5)	1402 (15.1)	0.010
CV selective	567 (21.0)	1983 (21.4)	0.009	348 (12.9)	1404 (15.1)	0.065
Digoxin	334 (12.4)	979 (10.6)	0.057	334 (12.4)	979 (10.6)	0.057
Anticoagulants	221 (8.2)	555 (6.0)	0.086	174 (6.4)	519 (5.6)	0.036
Antiarrhythmic agents	132 (4.9)	310 (3.3)	0.078	193 (7.2)	390 (4.2)	0.127
Lipid-lowering agents						
Statins	469 (17.4)	1567 (16.9)	0.012	458 (17.0)	2,135 (23.0)	0.152
Others	218 (8.1)	853 (9.2)	0.040	133 (4.9)	635 (6.9)	0.082
COPD medications						
Inhaled corticosteroids	392 (14.5)	1411 (15.2)	0.020	432 (16.0)	1230 (13.3)	0.077
LABA	250 (9.3)	801 (8.6)	0.022	341 (126)	1002 (10.8)	0.057
LAMA	58 (2.2)	202 (2.2)	0.002	103 (3.8)	206 (2.2)	0.093
Short-acting β_2_ agonists						
Nebulized	810 (30.0)	2174 (23.5)	0.148	1067 (39.5)	2583 (27.9)	0.249
Inhaled						
0 canister	2254 (83.5)	7768 (83.8)	0.008	2174 (80.5)	8051 (86.8)	0.171
≤ 6 canisters	381 (14.1)	1291 (13.9)	0.005	493 (18.3)	1158 (12.5)	0.160
> 6 canisters	65 (2.4)	213 (2.3)	0.007	33 (1.2)	63 (0.7)	0.056
Short-acting muscarinic antagonists						
Nebulized	674 (25.0)	1717 (18.5)	0.157	970 (35.9)	2365 (25.5)	0.227
Inhaled						
0 canister	2465 (91.3)	8567 (92.4)	0.040	2442 (90.4)	8814 (95.1)	0.179
≤ 6 canisters	205 (7.6)	610 (6.6)	0.040	246 (9.1)	433 (4.7)	0.176
> 6 canisters	30 (1.1)	95 (1.0)	0.008	12 (0.4)	25 (0.3)	0.029
Oral β_2_ agonists	1511 (56.0)	5586 (60.3)	0.087	1329 (49.2)	4279 (46.2)	0.062
Methylxanthines	1223 (45.3)	4600 (49.6)	0.086	1208 (44.7)	3783 (40.8)	0.080
Gastric acid suppressants						
H-blockers	1054 (39.0)	3338 (36.0)	0.063	851 (31.5)	2599 (28.0)	0.076
PPI	414 (15.3)	1006 (10.9)	0.133	454 (16.8)	976 (10.5)	0.184
Anti-inflammatory agents						
NSAID	1953 (72.3)	6998 (75.5)	0.072	1309 (48.5)	5117 (55.2)	0.135
Systemic corticosteroid	1593 (59.0)	5449 (58.8)	0.005	1570 (58.2)	4213 (45.4)	0.256
Aspirin (≥ 325 mg)	521 (19.3)	1722 (18.6)	0.018	387 (14.3)	1103 (11.9)	0.072
Psychotropic drugs						
Benzodiazepines and Z-drugs	1785 (66.1)	6134 (66.2)	0.001	1478 (54.7)	4807 (51.8)	0.058
Antipsychotics	611 (22.6)	1771 (19.1)	0.087	518 (19.2)	1529 (16.5)	0.070
Opioids	1443 (53.4)	5132 (55.4)	0.038	1092 (40.4)	3535 (38.1)	0.047
Vaccines (influenza and pneumonia)	1061 (39.3)	3682 (39.7)	0.008	798 (29.6)	3195 (34.5)	0.105

SD, standardized difference; s.d., standard deviation; COPD, chronic obstructive pulmonary disease; DM, diabetes mellitus; aDCSI, adapted diabetes complications severity index; NA, not applicable; No., number; ER, emergency room; OPD, outpatient department; CV, cardiovascular; GERD, gastroesophageal reflux disease; LABA, inhaled long-acting β_2_ agonists; LAMA, inhaled long-acting antimuscarinic antagonists; PPI, proton pump inhibitor; NSAID, nonsteroidal anti-inflammatory drug

^a^ All comorbidities, DM severity indicators, COPD severity indicators, and co-medications were measured in the year before the cohort entry date

^b^ SD with > 0.1 represents meaningful differences between two groups

^c^ All comorbidities, COPD severity indicators, aDSCI metabolic acidosis, and pneumonia and influenza vaccinations were measured in the year preceding the index date; co-medications and other DM severity indicators were measured in the 6 months before the index date

^d^ Regimens of oral hypoglycemic agents were measured at the cohort entry date, and the measurements during follow-up were similar to those of co-medications

^e^ Number of inpatient visit for COPD exacerbations was measured during 31–365 days before the cohort entry date

^f^ OPD visits for COPD exacerbations were defined as patients with a primary diagnosis of COPD refilling oral corticosteroids or antibiotics for use 3–14 days after an outpatient visit

Table 2 presents the associations between current use of each type of OHA and risk of severe COPD exacerbation. Current use of metformin versus that of other OHAs yielded a 19% (adjusted odds ratio [aOR], 0.81; 95% CI 0.73–0.91) and a 15% (aOR, 0.85; 95% CI 0.75–0.95) decreased risk of severe COPD exacerbation when controlling for covariates with standardized differences greater than 0.1 (model 1) and further adjusting for diabetic medication regimens (model 2), respectively. In contrast, current use of other OHAs, like sulfonylureas or DPP-4 inhibitors, was not associated with the risk of severe COPD exacerbation.

**Table 2:** Crude and adjusted ORs of severe COPD exacerbation associated with current use of different class of oral hypoglycemic agents

	Cases (n = 2700)	Controls (n = 9272)	OR (95% CI)		
			Crude OR	Model 1 Adjusted OR^a^	Model 2 Adjusted OR^b^
Current use, no, (%)^c^					
Metformin	1105 (40.9)	4654 (50.2)	0.75 (0.68–0.84)	0.81 (0.73–0.91)^d^	0.85 (0.75–0.95)^d^
Non-metformin	741 (27.4)	2348 (25.3)	Reference	Reference	Reference
Sulfonylureas	1181 (43.7)	4772 (51.5)	0.83 (0.74–0.93)	0.93 (0.83–1.04)	0.98 (0.86–1.11)
Non-sulfonylureas	665 (24.6)	2230 (24.1)	Reference	Reference	Reference
α-Glucosidase inhibitors	199 (7.4)	774 (8.4)	0.96 (0.81–1.14)	0.97 (0.82–1.16)	0.98 (0.82–1.18)
Non-α-glucosidase inhibitors	1647 (61.0)	6228 (67.2)	Reference	Reference	Reference
TZDs	123 (4.6)	566 (6.1)	0.81 (0.66–0.99)	0.87 (0.71–1.08)	0.87 (0.69–1.10)
Non-TZDs	1723 (63.8)	6436 (69.4)	Reference	Reference	Reference
DPP-4 inhibitors	181 (6.7)	645 (7.0)	1.07 (0.89–1.29)	1.09 (0.90–1.34)	1.13 (0.92–1.40)
Non-DPP-4 inhibitors	1665 (61.7)	6357 (68.6)	Reference	Reference	Reference
Meglitinides	241 (8.9)	651 (7.0)	1.44 (1.23–1.69)	1.18 (0.998–1.40)	1.19 (1.00–1.41)
Non-meglitinides	1605 (59.4)	6351 (68.5)	Reference	Reference	Reference

OR, odds ratio; COPD, chronic obstructive pulmonary disease; CI confidence interval; No., Number; TZD, thiazolidinedione; DPP-4, dipeptidyl peptidase-4; DM, diabetic mellitus

^a^ Adjusted for all covariates with standardized difference > 0.1 in Table 1

^b^ Adjusted for all covariates with standardized difference > 0.1 in Table 1 and DM therapy regimen in the 180 days prior to the index date

^c^ Current use was defined as the end date of the most recent prescription falling in the 30 days prior to the index date

^d^ *p* < 0.05

The associations between current use of each type of OHA in varying durations of therapy and the risk of severe COPD exacerbation are presented in Table 3. Current use of metformin versus that of other OHAs during the first 30 days of therapy carried a 1.55-fold (aOR, 1.55; 95% CI 1.28–1.88) increased risk of COPD severe exacerbation, even when further adjusting for diabetic medication regimens (aOR, 1.61; 95% CI 1.32–1.69), whereas a 28% (aOR, 0.72; 95% CI 0.58–0.89), 37% (aOR, 0.63; 95% CI 0.51–0.77), and 32% (aOR, 0.68; 95% CI 0.57–0.79) lower risk of severe COPD exacerbation was observed for 91–180 days, 181–365 days, and more than 365 days of metformin therapy, respectively. Similarly, current use of sulfonylurea for more than 90 days led to an approximately 30% reduced risk of severe COPD exacerbation, although an increased risk of severe COPD exacerbation was observed within the first 30 days of sulfonylurea use. Furthermore, current use of TZD had a similar duration effect as the use of metformin and sulfonylurea.

**Table 3:** Crude and adjusted ORs of severe COPD exacerbation associated with varying durations of current use of oral hypoglycemic agents

	Cases (n = 2700)	Controls (n = 9272)	OR (95% CI)		
			Crude OR	Model 1 Adjusted OR^a^	Model 2 Adjusted OR^b^
Current use, no (%)^c^					
Metformin					
≤ 30 days	222 (8.2)	472 (5.1)	1.51 (1.26–1.82)^d^	1.55 (1.28–1.88)^d^	1.61 (1.32–1.96)^d^
31–90 days	227 (8.4)	709 (7.7)	1.06 (0.89–1.26)	1.09 (0.91–1.31)	1.13 (0.94–1.37)
91–180 days	148 (5.5)	736 (7.9)	0.66 (0.54–0.80)^d^	0.69 (0.56–0.85)^d^	0.72 (0.58–0.89)^d^
181–365 days	167 (6.2)	964 (10.4)	0.54 (0.45–0.66)^d^	0.60 (0.50–0.73)^d^	0.63 (0.51–0.77)^d^
> 365 days	341 (12.6)	1773 (19.1)	0.58 (0.50–0.67)^d^	0.65 (0.56–0.76)^d^	0.68 (0.57–0.79)^d^
Non-metformin	741 (27.4)	2348 (25.3)	Reference	Reference	Reference
* p* for trend			< 0.001	< 0.001	< 0.001
Sulfonylureas					
≤ 30 days	220 (8.2)	372 (4.0)	2.02 (1.67–2.45)^d^	2.24 (1.83–2.75)^d^	2.35 (1.90–2.89)^d^
31–90 days	243 (9.0)	642 (6.9)	1.33 (1.11–1.59)^d^	1.46 (1.21–1.75)^d^	1.52 (1.26–1.85)^d^
91–180 days	137 (5.1)	757 (8.2)	0.63 (0.51–0.77)^d^	0.68 (0.55–0.84)^d^	0.72 (0.58–0.90)^d^
181–365 days	184 (6.8)	1023 (11.0)	0.60 (0.49–0.72)^d^	0.67 (0.55–0.81)^d^	0.70 (0.57–0.85)^d^
> 365 days	397 (14.7)	1978 (21.3)	0.64 (0.55–0.74)^d^	0.73 (0.63–0.85)^d^	0.76 (0.65–0.90)^d^
Non-sulfonylureas	665 (24.6)	2230 (24.1)	Reference	Reference	Reference
* p* for trend			< 0.001	< 0.001	< 0.001
Current use, no (%)^c^					
α-Glucosidase inhibitors					
≤ 30 days	42 (1.6)	135 (1.5)	1.14 (0.80–1.64)	1.12 (0.77–1.62)	1.12 (0.77–1.64)
31–90 days	48 (1.8)	175 (1.9)	1.04 (0.75–1.45)	1.04 (0.74–1.46)	1.05 (0.74–1.48)
91–180 days	26 (1.0)	120 (1.3)	0.80 (0.52–1.24)	0.77 (0.49–1.22)	0.78 (0.49–1.24)
181–365 days	30 (1.1)	133 (1.4)	0.85 (0.56–1.27)	0.84 (0.55–1.29)	0.85 (0.55–1.31)
> 365 days	53 (2.0)	211 (2.3)	0.94 (0.68–1.28)	1.02 (0.74–1.41)	1.03 (0.74–1.43)
Non-α-glucosidase inhibitors	1647 (61.0)	228 (67.2)	Reference	Reference	Reference
* p* for trend			0.525	0.810	0.835
TZDs					
≤ 30 days	31 (1.2)	74 (0.8)	1.63 (1.06–2.50)^d^	1.59 (1.02–2.49)^d^	1.59 (1.01–2.50)^d^
31–90 days	26 (1.0)	79 (0.9)	1.26 (0.81–1.98)	1.31 (0.82–2.09)	1.33 (0.83–2.14)
91–180 days	23 (0.9)	95 (1.0)	0.88 (0.55–1.41)	0.94 (0.58–1.54)	0.94 (0.57–1.55)
181–365 days	16 (0.6)	138 (1.5)	0.44 (0.26–0.75)^d^	0.52 (0.30–0.89)^d^	0.52 (0.30–0.89)^d^
> 365 days	27 (1.0)	180 (1.9)	0.53 (0.35–0.80)^d^	0.58 (0.37–0.89)^d^	0.57 (0.37–0.89)^d^
Non-TZDs	1723 (63.8)	6436 (69.4)	Reference	Reference	Reference
* p* for trend			0.004	0.050	0.039
Current use, no (%)^c^					
DPP-4 inhibitors					
≤ 30 days	32 (1.2)	95 (1.0)	1.31 (0.86–2.02)	1.27 (0.81–1.99)	1.31 (0.83–2.08)
31–90 days	44 (1.6)	128 (1.4)	1.29 (0.90–1.85)	1.26 (0.86–1.84)	1.30 (0.89–1.91)
91–180 days	36 (1.3)	107 (1.2)	1.33 (0.90–1.97)	1.42 (0.95–2.12)	1.47 (0.97–2.21)
181–365 days	28 (1.0)	127 (1.4)	0.83 (0.54–1.27)	0.82 (0.52–1.28)	0.85 (0.54–1.33)
> 365 days	41 (1.5)	188 (2.0)	0.81 (0.57–1.16)	0.89 (0.61–1.29)	0.93 (0.64–1.35)
Non-DPP-4 inhibitors	1665 (61.7)	6357 (68.6)	Reference	Reference	Reference
* p* for trend			0.586	0.787	0.860
Meglitinides					
≤ 30 days	54 (2.0)	86 (0.9)	2.36 (1.66–3.37)^d^	2.01 (1.39–2.92)^d^	2.03 (1.40–2.95)^d^
31–90 days	55 (2.0)	136 (1.5)	1.60 (1.16–2.21)	1.30 (0.92–1.83)	1.32 (0.93–1.88)
91–180 days	37 (1.4)	121 (1.3)	1.18 (0.81–1.72)	0.81 (0.54–1.21)	0.81 (0.54–1.22)
181–365 days	39 (1.4)	115 (1.2)	1.33 (0.91–1.93)	1.14 (0.77–1.70)	1.14 (0.77–1.69)
> 365 days	56 (2.1)	193 (2.1)	1.15 (0.85–1.57)	1.01 (0.74–1.39)	1.02 (0.74–1.40)
Non-meglitinides	1605 (59.4)	6351 (68.5)	Reference	Reference	Reference
* p* for trend			0.015	0.615	0.588

OR, odds ratio; COPD, chronic obstructive pulmonary disease; CI confidence interval; No., Number; TZD, thiazolidinedione; DPP-4, dipeptidyl peptidase-4; DM, diabetic mellitus

^a^ Adjusted for all covariates with standardized difference > 0.1 in Table 1

^b^ Adjusted for all covariates with standardized difference > 0.1 in Table 1 and DM therapy regimen in the 180 days prior to the index date

^c^ Current use was defined as the end date of the most recent prescription within the 30 days prior to the index date

^d^ *p* < 0.05

Table 4 presents the dose effects of current use of OHAs on severe COPD exacerbation. Despite a 19%-22% (model 1: aOR, 0.78; 95% CI 0.69–0.88; model 2: aOR, 0.81; 95% CI 0.71–0.92) decreased risks of severe COPD exacerbation with use of metformin at a daily dose ≤ 0.50 DDD, the reduced risk was absent with 0.51–1 DDD and > 1 DDD of metformin therapy. A similar dose effect was also observed with other types of OHAs, such as sulfonylureas, TZDs, and meglitinides. However, there was no statistically significant linear trend in the risk of severe COPD exacerbation from the lowest to highest categories of daily dose of antidiabetic medications.

**Table 4:** Crude and adjusted ORs of severe COPD exacerbation associated with use of oral hypoglycemic agents at different daily dose among current users

	Cases (n = 2700)	Controls (n = 9272)	OR (95% CI)		
			Crude OR	Model 1 Adjusted OR^a^	Model 2 Adjusted OR^b^
Current use, no (%)^c^					
Metformin					
≤ 0.5 DDD	649 (24.0)	2811 (30.3)	0.73 (0.65–0.82)^d^	0.78 (0.69–0.88)^d^	0.81 (0.71–0.92)^d^
0.51–1 DDD	391 (14.5)	1601 (17.3)	0.78 (0.67–0.89)^d^	0.85 (0.73–0.98)^d^	0.89 (0.76–1.04)
> 1 DDD	65 (2.4)	242 (2.6)	0.86 (0.64–1.15)	1.05 (0.78–1.42)	1.10 (0.81–1.50)
Non-metformin	741 (27.4)	2348 (25.3)	Reference	Reference	Reference
* P* for trend			0.002	0.084	0.358
Sulfonylureas					
≤ 0.5 DDD	325 (12.0)	1170 (12.6)	0.92 (0.79–1.07)	0.97 (0.82–1.13)	1.00 (0.85–1.18)
0.51–1 DDD	389 (14.4)	1481 (16.0)	0.90 (0.78–1.04)	1.00 (0.86–1.16)	1.05 (0.90–1.23)
> 1 DDD	467 (17.3)	2121 (22.9)	0.73 (0.64–0.84)^d^	0.85 (0.74–0.98)^d^	0.90 (0.77–1.04)
Non-sulfonylureas	665 (24.6)	2230 (24.1)	Reference	Reference	Reference
* P* for trend			< 0.001	0.177	0.607
Current use, no (%)^c^					
α-Glucosidase inhibitors					
≤ 0.5 DDD	159 (5.9)	649 (7.0)	0.93 (0.77–1.12)	0.93 (0.77–1.13)	0.94 (0.77–1.15)
0.51–1 DDD	37 (1.4)	120 (1.3)	1.09 (0.74–1.59)	1.17 (0.78–1.75)	1.17 (0.78–1.77)
> 1 DDD	3 (0.1)	5 (0.1)	1.80 (0.41–7.93)	1.73 (0.37–8.18)	1.73 (0.36–8.19)
Non-α-glucosidase inhibitors	1647 (61.0)	6228 (67.2)	Reference	Reference	Reference
* P* for trend			0.837	0.550	0.550
TZDs					
≤ 0.5 DDD	22 (0.8)	126 (1.4)	0.63 (0.39–0.998)^d^	0.64 (0.40–1.04)	0.65 (0.40–1.05)
0.51–1 DDD	93 (3.4)	397 (4.3)	0.89 (0.70–1.12)	0.98 (0.76–1.25)	0.97 (0.75–1.26)
> 1 DDD	8 (0.3)	43 (0.5)	0.65 (0.30–1.40)	0.67 (0.29–1.55)	0.66 (0.28–1.55)
Non-TZDs	1723 (63.8)	6436 (69.4)	Reference	Reference	Reference
* P* for trend			0.217	0.816	0.818
Current use, no (%)^c^					
DPP-4 inhibitors					
≤ 0.5 DDD	50 (1.9)	180 (1.9)	1.07 (0.77–1.50)	1.05 (0.74–1.48)	1.07 (0.75–1.53)
0.51–1 DDD	117 (4.3)	431 (4.7)	1.03 (0.82–1.29)	1.09 (0.86–1.38)	1.14 (0.89–1.46)
> 1 DDD	14 (0.5)	34 (0.4)	1.58 (0.82–3.06)	1.36 (0.68–2.71)	1.43 (0.71–2.85)
Non-DPP-4 inhibitors	1665 (61.7)	6357 (68.6)	Reference	Reference	Reference
* P* for trend			0.497	0.568	0.471
Meglitinides					
≤ 0.5 DDD	86 (3.2)	238 (2.6)	1.37 (1.06–1.77)^d^	1.06 (0.81–1.39)	1.05 (0.80–1.38)
0.51–1 DDD	116 (4.3)	286 (3.1)	1.59 (1.27–2.00)^d^	1.34 (1.05–1.70)^d^	1.35 (1.06–1.71)^d^
> 1 DDD	39 (1.4)	127 (1.4)	1.24 (0.86–1.78)	1.07 (0.73–1.57)	1.10 (0.75–1.62)
Non-meglitinides	1605 (59.4)	6351 (68.5)	Reference	Reference	Reference
* P* for trend			< 0.001	0.077	0.065

OR, odds ratio; COPD, chronic obstructive pulmonary disease; CI confidence interval; No., Number; DDD, defined daily dose; TZD, thiazolidinedione; DPP-4, dipeptidyl peptidase-4; DM, diabetic mellitus

^a^ Adjusted for all covariates with standardized difference > 0.1 in Table 1

^b^ Adjusted for all covariates with standardized difference > 0.1 in Table 1 and DM therapy regimen in the 180 days prior to the index date

^c^ Current use was defined as the end date of the most recent prescription within the 30 days prior to the index date

^d^ *p* < 0.05

Table 5 indicates that patients currently receiving any type of oral hypoglycemic monotherapy did not present any change in the risk of severe COPD exacerbation compared with those presently using other classes of monotherapy. In contrast, dual therapy with metformin and sulfonylureas yielded a 22% (aOR, 0.78; 95% CI 0.64–0.95) reduction in the exacerbation risk as compared with other therapies comprising two or more components (Table 6).

**Table 5:** Crude and adjusted ORs of severe COPD exacerbations associated with the current receipt with each type of oral hypoglycemic agent in monotherapy

	Cases (n = 2700)	Controls (n = 9272)	Crude OR (95% CI)	Adjusted OR^a^ (95% CI)
Current use of monotherapy, no (%)^b^				
Metformin	346 (12.8)	1242 (13.4)	0.85 (0.73–0.99)^d^	0.87 (0.74–1.02)
Sulfonylureas	423 (15.7)	1344 (14.5)	1.08 (0.93–1.25)	1.14 (0.97–1.34)
α-Glucosidase inhibitors	34 (1.3)	139 (1.5)	0.77 (0.53–1.14)	0.74 (0.50–1.12)
TZDs	8 (0.3)	33 (0.4)	0.82 (0.38–1.79)	0.84 (0.37–1.89)
DPP-4 inhibitors	36 (1.3)	74 (0.8)	1.59 (1.04–2.41)^d^	1.46 (0.94–2.27)
Meglitinides	96 (3.6)	252 (2.7)	1.26 (0.98–1.62)	1.05 (0.80–1.36)
Other classes of monotherapy^c^			Reference	Reference

Abbreviations: OR, odds ratio; COPD, chronic obstructive pulmonary disease; CI confidence interval; No., Number; TZD, thiazolidinedione; DPP-4, dipeptidyl peptidase-4

^a^ Adjusted for all covariates with standardized difference > 0.1 in Table 1

^b^ Current use was defined as the end date of the most recent prescription within the 30 days prior to the index date

^c^ Each antidiabetic agent monotherapy was separately compared with all of the other types of antidiabetic agents monotherapy combined

^d^ *p* < 0.05

**Table 6:** Crude and adjusted ORs of severe COPD exacerbation associated with dual or more therapies of oral hypoglycemic agents

	Cases (n = 2700)	Controls (n = 9272)	Crude OR (95% CI)	Adjusted OR^a^ (95% CI)
Current use of dual or more therapy, no (%)^b^				
Metformin + sulfonylureas	460 (17.0)	2,238 (24.1)	0.71 (0.59–0.86)^c^	0.78 (0.64–0.95)^c^
Metformin + α-glucosidase inhibitors	12 (0.4)	51 (0.6)	1.10 (0.57–2.09)	0.93 (0.47–1.81)
Metformin + TZDs	5 (0.2)	33 (0.4)	0.62 (0.24–1.63)	0.71 (0.27–1.88)
Metformin + DPP-4 inhibitors	22 (0.8)	76 (0.8)	1.25 (0.77–2.05)	1.30 (0.78–2.16)
Metformin + meglitinides	46 (1.7)	132 (1.4)	1.52 (1.07–2.16)^c^	1.32 (0.91–1.91)
Other combinations of dual therapy	118 (4.4)	402 (4.3)	1.36 (1.08–1.71)^c^	1.27 (0.997–1.61)
All triple therapy	235 (8.7)	960 (10.4)	1.07 (0.91–1.27)	1.12 (0.94–1.34)
Metformin-contained triple therapy	214 (7.9)	882 (9.5)	1.08 (0.90–1.28)	1.13 (0.94–1.35)
Other triple therapy	21 (0.8)	78 (0.8)	1.10 (0.67–1.81)	1.09 (0.65–1.84)
Other types of dual or more therapy^d^			Reference	Reference

OR, odds ratio; COPD, chronic obstructive pulmonary disease; CI confidence interval; No., Number; TZD, thiazolidinedione; DPP-4, dipeptidyl peptidase-4

^a^ Adjusted for all covariates with standardized difference > 0.1 in Table 1

^b^ Current use was defined as the end date of the most recent prescription within the 30 days prior to the index date

^c^ *p* < 0.05

^d^ Each of the dual or more therapy of antidiabetic agents was separately compared with all of the other types of dual or more therapy of antidiabetic agents combined

**Fig. 2:**
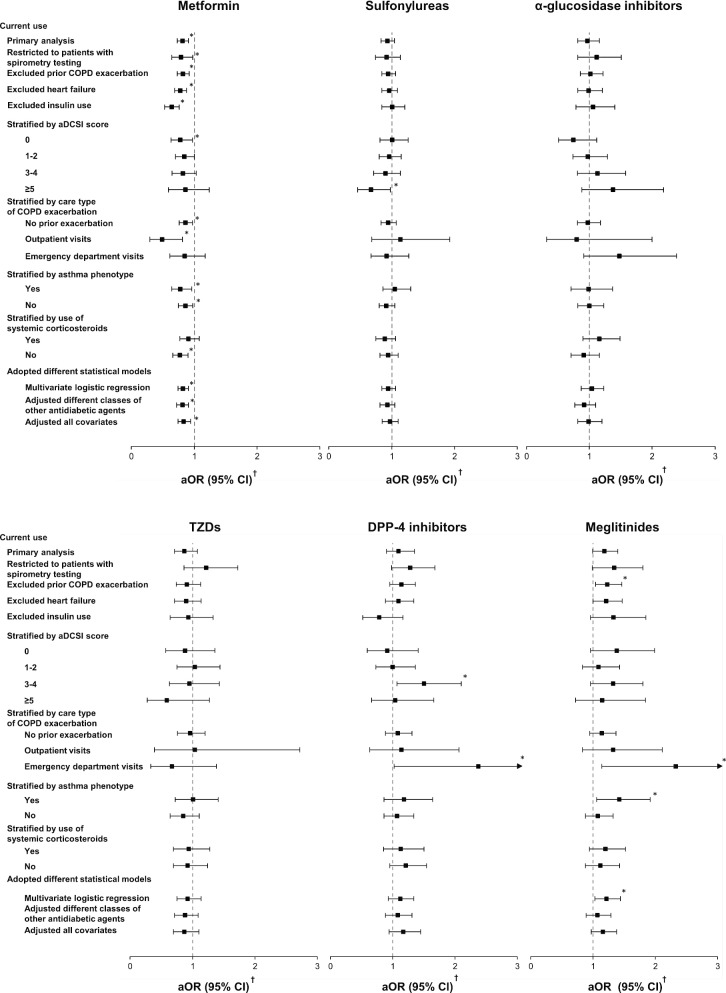
Sensitivity analyses and subgroup analyses for the risk of severe COPD exacerbation with current use of oral hypoglycemic agents. COPD, chronic obstructive pulmonary disease; aDCSI, adapted diabetes complications severity index; aOR, adjusted odds ratio. *p < 0.05. ^†^Adjusted for all covariates with standardized difference > 0.1 in Table 1

**Fig. 3:**
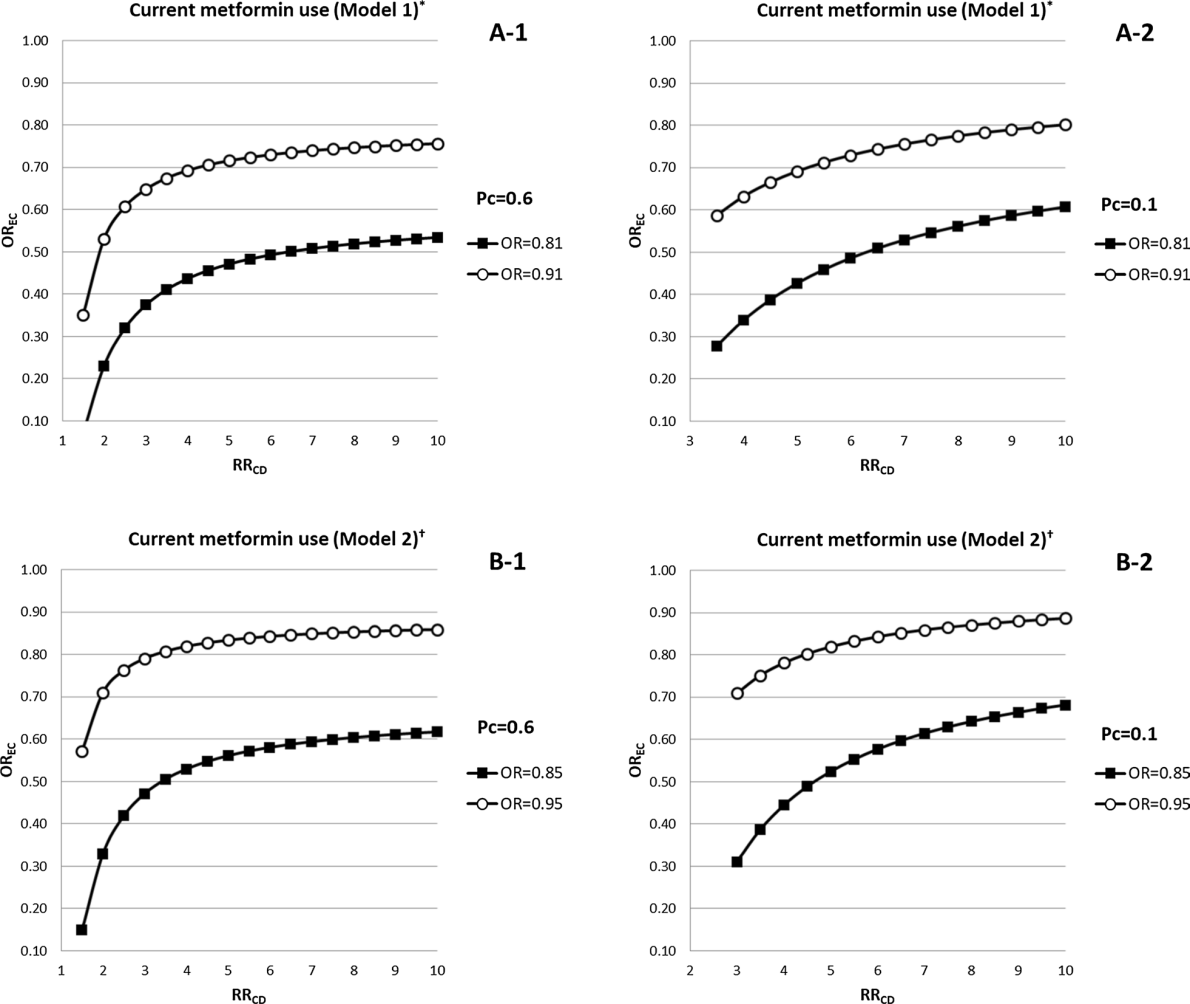
The impact of an unmeasured confounder on the current use of metformin examined by using the rule-out approach. We assumed that the prevalence of an unmeasured confounder was 60% in **a-1** and **b-1**, as well as 10% in **a-2** and **b-2**, respectively. The prevalence of current use of metformin was estimated to be approximately 50% in the study cohort. In all panels, the combinations of RR_CD_ and OR_EC_ that lie on the line or in the lower right zone of each line indicate an unmeasured confounder could account for the reduced risk of severe COPD exacerbation from current use of metformin among diabetic patients with COPD (Model 1: adjusted RR = 0.81, the upper 95% confidence limit = 0.91; Model 2: adjusted RR = 0.85, the upper 95% confidence limit = 0.95). OR, odds ratio; RR_CD_, risk of severe COPD exacerbation from an unmeasured confounder; OR_EC_, odds ratio between current metformin use and an unmeasured confounder; P_c_, the prevalence of an unmeasured confounder in this study. *In model 1, we adjusted for all covariates with standardized difference > 0.1 in Table 1. ^†^In model 2, we adjusted for all covariates with standardized difference > 0.1 in Table 1 and DM therapy regimen in the 180 days prior to the index date

Overall, the main findings were robust in most of the sensitivity analyses (Fig. 2). The findings regarding the decreased risks of severe COPD exacerbation with the current use of metformin were replicated when restricted to patients who had undergone spirometry testing, to those without severe COPD exacerbation at baseline, to patients without heart failure and use of insulin, and when adopting different statistical models. The observed protective effects of metformin were primarily confined to patients with outpatient-related or no COPD exacerbations during follow-up. When stratified by use of systemic corticosteroids, the reduced risk of severe COPD exacerbation with current metformin use was confined to those without prior systemic corticosteroid use. Figure 3 indicates that an unmeasured confounder needs to be 2 times less prevalent in metformin users then in non-metformin users and to increase the risk of severe COPD exacerbation by more than three to sixfold to completely explain the observed association between current use of metformin and risk of severe COPD exacerbation.

## Discussion

This DRS-matched nested case–control study of more than 23,000 patients with COPD diagnosed with DM revealed a significantly decreased risk (15–20%) of severe COPD exacerbation associated with current use of metformin versus that of other OHAs. This observed reduced risk with metformin therapy persisted in patients who had undergone spirometry testing, had no previous severe COPD exacerbation or heart failure, and when performing different statistical adjustment models. Additionally, current use of metformin, sulfonylureas, and TZDs lasting for > 90 days, respectively, was associated with an approximately 30–40% reduction in the risk of severe COPD exacerbation. Overall, we provided the first evidence that using certain types of OHAs, including metformin, sulfonylureas and TZD, is associated with a duration-dependent decreased risk of severe COPD exacerbation in patients with COPD and DM.

Partly, our findings agree with those of two previous reports [16, 17] that only examined the association between metformin or TZD use and COPD exacerbation, and our remaining findings provide new information. A cohort study of 11,260 US Medicare patients with low COPD complexity diagnosed with DM observed a 34% (aOR, 0.66; 95% CI 0.52–0.85) reduced risk of COPD-specific ER visits or hospitalizations with the use of metformin [16]. However, this study could have been threatened by immortal time bias, misclassification of drug exposure during follow-up, and selection bias. Moreover, the findings revealed a reduced COPD exacerbation risk when assessing the effects of metformin therapies lasting for > 90 days, similar to our findings. Assessment of a longer duration of treatment with metformin may be the main driver of the consistent findings of the two studies. Another cohort study of 50,243 US veterans reported a 19% (incidence rate ratio, 0.81; 95% CI 0.68–0.97) reduced risk of hospitalization for COPD exacerbations with TZD use [17], which is much lower than that observed with TZD therapy lasting for > 180 days in our study. The findings by Rinne et al., however, had several study limitations. First, the average duration of TZD therapy was undefined in Rinne’s study [17]. Second, TZD users could receive other antidiabetic medications [17], which were not addressed. On the other hand, our findings regarding the beneficial effect of sulfonylureas on COPD; the effects of α-glucosidase inhibitors, DPP-4 inhibitors, and meglitinides; and the reported duration-effects provide novel insights into the association between use of antidiabetic agents and risk of severe COPD exacerbation.

Despite not measuring COPD exacerbation, recent studies have revealed potentially beneficial effects with use of several types of antidiabetic agents in patients with COPD and DM [28]. Although DM is reported to have a negative impact on COPD, one study has found that COPD patients co-diagnosed with DM did not have an impaired pulmonary capillary function, such as the diffusing capacity of carbon monoxide, compared to patients with COPD only [29]. This phenomenon could probably be attributable to the systemic anti-inflammatory effects from use of antidiabetic medications in COPD patients [29]. Additionally, multiple studies have reported that use of insulin sensitizers, such as metformin and TZD in COPD may improve lung functions and reduce risks of all-cause mortality and lung cancer [30–32]. Collectively, our data collide with the abovementioned studies indicating that patients with COPD and DM may benefit from use of antidiabetic medications in several important clinical outcomes.

Several potentially biological plausibilities may exist for the observed protective effect of metformin against severe COPD exacerbation. First, metformin exerts an anti-inflammatory effect by activating adenosine monophosphate-activated protein kinase [33], which could inhibit the pro-inflammatory and inflammatory responses in vascular endothelial and smooth muscle cells [34, 35] and generate anti-atherosclerotic effects by reducing inflammatory cell adhesion to the blood vessel endothelium [36]. Additionally, metformin used for 3 months significantly reduced the level of inflammatory markers ICAM-1, TNF-a, and IL-6 in patients with drug-naïve type II DM [37]. Second, metformin could improve respiratory muscle function, which may alleviate COPD symptom worsening. Specifically, Six-month metformin treatment significantly increased (11%) inspiratory muscle strength in patients with moderate-to-severe COPD diagnosed with DM [30], although its clinical significance is unclear owing to lack of clinical improvement in inspiratory muscle strength among patients with COPD. Third, mitigation of insulin resistance with metformin use could be another potential explanation. Chronic systemic inflammation in COPD is highly associated with insulin resistance [15, 38], which further impairs airway obstruction and increases COPD exacerbation occurrence [39, 40]. Therefore, as metformin effectively attenuates insulin resistance, it may benefit COPD exacerbation.

The observed beneficial effect of sulfonylureas on severe COPD exacerbation may result more from the drug’s potential influence on hypoxia-induced insulin resistance than from its anti-inflammatory effect, despite scant evidence. In vitro and in vivo studies have shown that K_ATP_ channel-specific sulfonylureas counteract the inflammatory effects of K_ATP_ channel stimulation via mitogen-activated protein kinase pathways in monocytes and macrophages [41]. Nevertheless, in humans, there are no documented decreased levels of systemic inflammatory biomarkers with the use of K_ATP_ channel-specific sulfonylureas glyburide [42], glimepiride [43], and gliclazide [44]. Conversely, the reduced hypoxia-induced insulin resistance from sulfonylureas could potentially act as an alternative mechanism. Chronic intermittent hypoxia could lead to insulin resistance, impair glucose tolerance, and consequently worsen COPD [45]. One animal study has reported that the sulfonylurea tolbutamide reversed the anoxic activation of K_ATP_ channels in the dorsal vagal neurons of mouse brainstem slices [46]. Sulfonylureas could indirectly improve pulmonary function by reducing hypoxia-induced insulin resistance.

There are several possible explanations for the observed increased risk of severe COPD exacerbation within the first 30 days after treatment with several OHA classes, especially sulfonylureas and meglitinide. A long time is probably required for the aforementioned mechanisms underlying the beneficial effect of OHAs on COPD exacerbation to kick in. For instance, at least 3-month treatment of metformin is needed to reduce inflammatory biomarker levels [37]. Therefore, patients with COPD receiving a short-term treatment with OHAs could not immediately attain the benefits against COPD progression. Alternatively, confounding by indication bias could contribute to our findings because uncontrolled hyperglycemia is associated with COPD exacerbation [40, 47] and a substantial proportion of patients initially receiving OHAs may have continuing, impaired fasting glucose and non-targeted hemoglobin A1c levels. Therefore, the observed increased risk during the first 30 days after using OHAs could simply result from an uncontrolled glucose level. If this bias truly exists, using all classes of OHAs should have led to similar positive results, which was not the case for α-glucosidase inhibitors and DPP-4 inhibitors in our study. Moreover, the adoption of the active-comparator design in our study may ease the concern about the presence of this bias. Another cause for the findings could be the initial OHA use for hyperglycemia induced by systemic corticosteroids [48, 49], which are most frequently prescribed for COPD worsening in outpatient settings that could progress to severe exacerbation requiring hospitalization [6]. Considering this, and given that sulfonylureas and meglitinide are the recommended therapies for corticosteroid-induced hyperglycemia [49], we could have observed a higher increased risk of COPD-related hospitalization within the first 30 days after the initial use of sulfonylureas and meglitinide.

Several attributes of our findings are pivotal from a clinical point of view. Our evidence on the reduction in the risk of severe COPD exacerbation following treatment with specific types of OHAs should be incorporated into the benefit-and-risk evaluation of pharmacological treatments for management of DM in patients with COPD. In the presence of COPD comorbidity, the use of OHAs needs to be considered according to their impact on COPD and antihyperglycemic effects. According to our findings, we suggest metformin as the first consideration for managing DM in patients with COPD if they have no contraindication for the drug. This recommendation is based on the evidence from the current treatment guidelines of DM management, suggesting metformin as the first-line therapy for patients with diabetes if tolerated and according to our real-world evidence on the reduced severe COPD exacerbation with metformin use. Under similar considerations, sulfonylureas may be considered as the preferred second-line therapy for patients in whom targeted glycemia levels have not been reached, for those intolerant to metformin, and for those not prone to sulfonylurea-induced adverse drug reactions, such as adverse hypoglycemia and cardiovascular events. According to our main findings and the fact that metformin has been reported as a safe medication in non-diabetic patients, such as polycystic ovary syndrome patients [50], well-designed randomized controlled trials are urgently required to confirm the observed beneficial effects of metformin in preventing COPD exacerbations.

This is the first large observational study that systemically assessed the effects of all classes of OHAs on severe COPD exacerbation in a nationwide population with COPD and DM. We also found that the timing and duration of oral antihyperglycemic therapy for management of DM play a pivotal role in protecting against severe COPD exacerbation. Furthermore, adopting a nested case–control design allowed the examination of numerous severe COPD exacerbation events. Moreover, employing a DRS-matching scheme could effectively minimize the impact of confounding and selection bias on our findings. As this study measured hard endpoints (severe COPD exacerbation requiring hospitalization), our findings could more closely reflect real-world conditions compared with those of previous studies that evaluated the impact of OHAs on changes in inflammatory biomarker levels or lung function data.

Our findings, however, should be interpreted with several limitations. First, as there are few imbalanced covariates between cases and controls, selection bias could not be entirely ruled out. To address this, we performed the DRS-matched process, adopted active-comparator analyses, and adjusted for all covariates with standard difference > 0.1. Second, the analyzed database lacked information on hemoglobin A1c, fasting glucose, forced expiratory volume in one second (FEV_1_), COPD Assessment Test (CAT) data, and modified Medical Research Council (mMRC) parameters; therefore, a confounding effect resulting from these unmeasured parameters is possible. Nevertheless, we considered comprehensive proxy indicators for both DM and COPD severities, which were quite well-balanced at baseline. Additionally, an unmeasured confounder is unlikely to fully explain our observed reduced risk of severe exacerbation associated with current use of metformin based on the rule-out analysis. Third, our study was not equipped with sufficient statistical power to examine the effect of the individual agents within each class of the antidiabetic medications on the risk of severe COPD exacerbations. Fourth, novel antidiabetic medications like sodium-glucose co-transporter 2 inhibitors and glucagon-like peptide-1 receptor agonists could not be assessed because of limited sizes. Fifth, we considered only severe COPD exacerbations as this severity of exacerbation had the most impact on morbidity and mortality; our findings could not be applied to patients with mild or moderate COPD exacerbations. Finally, our findings may be subject to the healthy user bias because patients could have been carefully monitored by healthcare professionals or intensively received respiratory medications during follow-up. However, all patients included in the current study were required to use both COPD and antidiabetic medications. If healthy user bias did exist, it would have an impact not only in case groups but also in control groups. Additionally, our observed reduced risk of COPD exacerbations was confined to specific types of antidiabetic medications as opposed to all classes of OHAs, which may preclude the presence of health user bias in the present study. Moreover, we have performed a disease-risk score matching approach and multivariable analysis to control for severity of COPD and DM as well as use of COPD medications at baseline and during follow-up, respectively. The rule-out analysis, however, pointed out that it is unlikely for unmeasured confounder, such as training in disease management, to fully account for our main findings.

## Conclusions

This large observational study of more than 23,000 COPD patients co-diagnosed with DM observed a duration-dependent beneficial effect of current use of metformin, sulfonylureas, and TZDs, respectively, on severe COPD exacerbation.

## Data Availability

The authors are restricted from sharing the analyzed data of current study because the availability of public access to the Taiwan National Health Insurance Research Database is forbidden by the current laws of Taiwan according to the regulations of the Ministry of Health and Welfare (https://dep.mohw.gov.tw/DOS/np-2497-113.html). To request access to the Taiwan National Health Insurance claims database, please contact the Health and Welfare Data Science Center, Ministry of Health and Welfare, Taiwan, ROC (https://dep.mohw.gov.tw/DOS/np-2497-113.html).

## Abbreviations

aOR: Adjusted odds ratio
COPD: Chronic obstructive pulmonary disease
DRS: Disease risk score
DM: Diabetes mellitus
DPP-4: Dipeptidyl peptidase-4
OHAs: Oral hypoglycemic agents
DDD: Defined daily dose
ER: Emergency room
ICD-9-CM: International Classification of Diseases, Ninth Revision, Clinical Modification
LGTD: Longitudinal Generation Tracking Database
OR: Odds ratios
TZD: Thiazolidinedione
